# Bilateral Symmetric Gastrocnemius Myositis Secondary to COVID-19

**DOI:** 10.7759/cureus.29127

**Published:** 2022-09-13

**Authors:** Aishwarya Sharma, Abhishek Matta, Dinesh Bande

**Affiliations:** 1 Medicine, University of North Dakota School of Medicine and Health Sciences, Grand Forks, USA; 2 Pulmonary and Critical Care Medicine, University of Texas Medical Branch at Galveston, Galveston, USA; 3 Internal Medicine, University of North Dakota School of Medicine and Health Sciences, Fargo, USA; 4 Internal Medicine, Sanford Health, Fargo, USA

**Keywords:** coronavirus disease 2019, gastrocnemius myositis, creatinine kinase, gastrocnemius, myositis, sars-cov-2 infection, covid-19

## Abstract

As the coronavirus disease 2019 (COVID-19) pandemic perseverates on, more insight into the multidisciplinary manifestations of COVID-19 is being brought to the forefront. Musculoskeletal presentations range from mild creatinine kinase elevation and myalgias to severe rhabdomyolysis. We present a case of a patient who presented with symmetric myositis of bilateral gastrocnemius muscles secondary group.

## Introduction

While coronavirus disease 2019 (COVID-19) was initially understood as an upper respiratory condition with characteristic symptoms such as fever, cough, and sore throat, published case reports are describing atypical presentations, which highlight the non-respiratory manifestations of this debilitating disease. The musculoskeletal system is no exception with isolated instances of COVID-19 manifestations ranging from elevated creatinine kinase (CK) to severe rhabdomyolysis [1]. This case report highlights the diagnostic challenge of COVID-19-related myositis and the subsequent need to raise physician awareness regarding severe rhabdomyolysis.

## Case presentation

A 35-year-old male with a past medical history significant for tobacco use, generalized anxiety disorder, and attention deficit hyperactivity disorder (ADHD) presented to the emergency department with a four-day history of severe bilateral leg pain, which impeded his weight-bearing activities. He denied any trauma, crush injury, or strenuous exercise. He reported no fever, chills, numbness, or tingling but extending his legs resulted in cramping and excruciating pain, which starts in his calves and occasionally radiates up to the posterior thigh. Lab work in the emergency department was significant for elevated CK, uric acid, C-reactive protein (CRP), erythrocyte sedimentation rate (ESR), and a positive COVID-19 polymerase chain reaction test (Table 1). The patient was not vaccinated against COVID-19.

**Table 1:** Lab work on admission to our facility. Bolded values indicate results outside of the corresponding reference range. Empty boxes indicate days when labs were not drawn.

Patient’s value	Day 1	Day 2	Day 3	Day 12	Reference range
Creatinine kinase	10,149	6,715	2,929	121	30-200 U/L
Uric acid	7.7				3.5-7.4 mg/dL
C-reactive protein	11				<5.0 mg/L
Erythrocyte sedimentation rate	18				0-14 mm/hour

**Figure 1:**
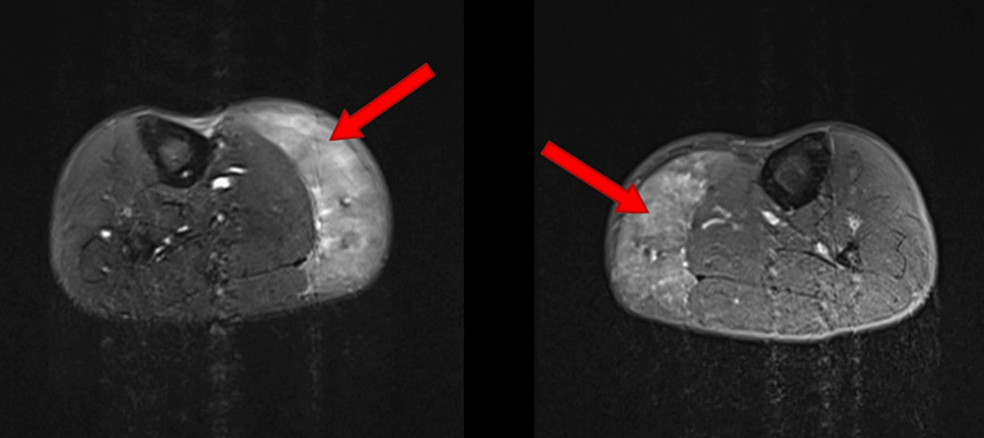
Right and left gastrocnemius axial view, respectively. Heterogeneous high T2 and short-TI inversion recovery signal throughout the gastrocnemius musculature symmetric compared to the opposite leg consistent with diffuse edema. Red arrows indicate areas of diffuse swelling.

**Figure 2:**
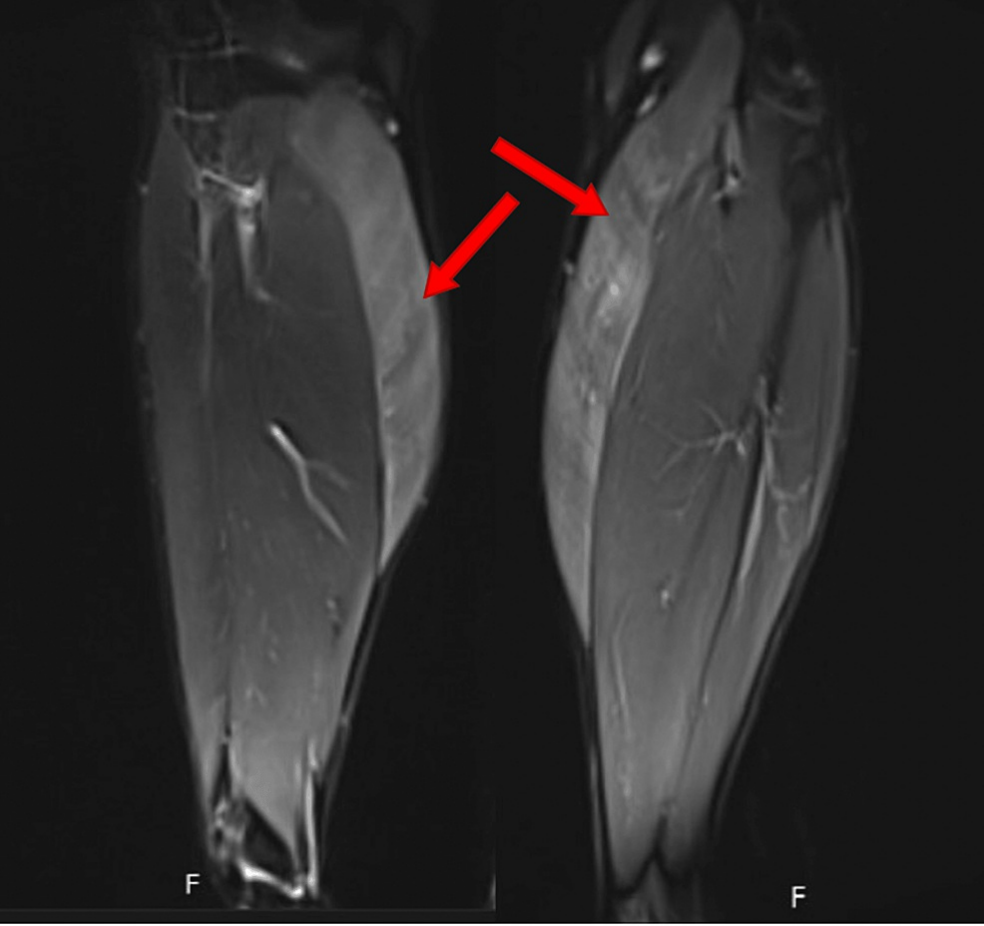
Right and left gastrocnemius coronal view, respectively. Red arrows indicate areas of diffuse edema.

X-ray films of bilateral tibia and fibula did not show any fractures, but MRI of the bilateral lower extremities was significant for symmetric diffuse edema of the medial gastrocnemius consistent with myositis of bilateral lower extremities as indicated by the red arrows in the figures provided (Figures 1, 2). Venous Doppler of the lower extremities ruled out deep venous thrombosis. The distal neurovascular bundle was intact, which ruled out compartment syndrome. Echo did not show any signs of myocarditis and troponin I was normal as well.

The patient was diagnosed with myositis secondary to COVID-19 and started on aggressive hydration to prevent acute kidney injury. CK levels down trended during the remainder of his hospital stay. An autoimmune myositis panel was obtained to rule out other etiologies for myositis, and it came back negative (Table 2). On day three, the patient experienced symptomatic improvement and was discharged home. The patient reported near complete resolution of symptoms during clinic follow-up a week after discharge.

**Table 2:** All values for the autoimmune panel were negative and within the reference range. MDA-5: anti-melanoma differentiation-associated gene 5 antibody; SRP: anti-signal recognition particle antibody; NXP-2: anti-nuclear matrix protein 2 antibody; U2 snRNP: U2 small nuclear ribonucleoprotein antibody; Anti-SS-A: anti-Sjogren’s-syndrome-related antigen A autoantibody.

	Reference range	Patient’s value
Anti-Jo-1 antibody	<20 units	<20
Anti-U1RNP antibody	<20 units	<20
MDA-5 (P140)(CADM-140)	<20	<20
SRP	Negative	Negative
NXP-2 (P140)	<20 units	<20
U2 snRNP	Negative	Negative
Anti-SS-A 52 kD ab, IgG	<20 units	<20

## Discussion

Isolated myositis as the only presentation of acute COVID-19 infection has been reported in very few cases [2]. To our knowledge, this is the only case of myositis with symmetric involvement of bilateral gastrocnemius muscles. Although the exact pathophysiology has not been elucidated, researchers have postulated that the virus triggers muscle inflammation via angiotensin-converting enzyme 2 (ACE2) receptor-mediated direct entry and injury to muscle fibers [3]. Direct invasion of viral antigens into myocytes results in structural deformity and clinical presentation of elevated CK and pain. SARS-CoV-2 may trigger hyperinflammation by binding toll-like receptor 4 and contribute to the T-cell expansion and an overall proinflammatory state with a rise in tumor necrosis factor (TNF)-alpha, interferon-alpha, interleukin-1, and interleukin-6 [4]. This upregulation of cytokines can subsequently cause muscle damage.

While muscle biopsies can offer a definitive diagnosis, their use remains limited, given the risk of extensive bleeding and increased recovery time [5,6]. MRIs remain a reliable, noninvasive option to detect muscle edema [7]. In our patient, elevated CK and inflammatory markers, negative autoimmune panel, and myositis workup led to the diagnosis of COVID-19-related myositis. To our knowledge, this is the first described case of a patient with symmetric involvement of muscle groups in extremities secondary to COVID-19-related myositis.

## Conclusions

Myositis is a rare manifestation of COVID-19. Given a wide range in CK levels and a lack of direct correlation in disease severity, physicians should work diligently to utilize elevated CK and inflammatory markers in addition to myositis-specific autoimmune panel and MRI to diagnose this infrequent, non-respiratory complication of COVID-19.
